# Editorial: Highlights in medical and surgical rehabilitation 2021/22

**DOI:** 10.3389/fresc.2023.1219924

**Published:** 2023-06-09

**Authors:** Areerat Suputtitada

**Affiliations:** ^1^ Department of Rehabilitation Medicine, Faculty of Medicine, Chulalongkorn University, Bangkok, Thailand; ^2^ Department of Rehabilitation Medicine, King Chulalongkorn Memorial Hospital, The Thai Red Cross Society, Bangkok, Thailand; ^3^ Excellence Center for Gait and Motion, King Chulalongkorn Memorial Hospital, The Thai Red Cross Society, Bangkok, Thailand; ^4^ Biomedical Engineering, Chulalongkorn University, Bangkok, Thailand; ^5^ Neurorehabilitation Research Unit, Faculty of Medicine, Chulalongkorn University, Bangkok, Thailand

**Keywords:** calcification and fibrosis removal, brain stimulation, tele-rehabilitation, long COVID, virtual reality, robotic, wearable technology, regenerative medicine

**Editorial on the Research Topic** Highlights in medical and surgical rehabilitation 2021/22

Rehabilitation is a crucial component of universal health coverage, which encompasses promoting good health, preventing disease, treatment, and palliative care. It helps individuals of all ages, from children to older people, to engage in everyday activities independently and participate in education, work, and recreation. Worldwide, approximately 2.4 billion people live with a health condition that could benefit from rehabilitation, and the demand for rehabilitation is predicted to rise due to changes in the population’s health and characteristics. Rehabilitation is essential in achieving Sustainable Development Goal 3: “Ensure healthy lives and promote well-being for all at all ages.” (1) Rehabilitation is personalized, with interventions that are targeted at individuals’ goals and preferences, such as speech and language therapy for those with brain lesions or exercise training for those with Parkinson’s disease. Rehabilitation can be provided in various settings, such as outpatient clinics, hospitals, and community settings. Rehabilitation’s benefits include reducing the impact of a wide range of health conditions, such as chronic diseases, cancers, and diabetes, and preventing complications associated with conditions such as spinal cord injury, stroke, or fractures. It also helps to minimize or slow down the disabling effects of chronic health conditions by equipping people with self-management strategies and assistive products. Rehabilitation is an investment with benefits for both individuals and society. It is essential to have timely, high-quality, and affordable rehabilitation interventions available to all, starting as early as possible. However, in some low- and middle-income countries, more than 50% of people do not receive the rehabilitation services they require. Rehabilitation is not only for people with disabilities or long-term or physical impairments; it is an essential health service for anyone with an acute or chronic health condition, impairment, or injury that limits functioning and, as such, should be guaranteed for anyone who needs it. Improving access to rehabilitation for all people is a constant challenge. It necessitates a multifaceted approach, including research into new techniques, rehabilitation methods, and care organization. By conducting research in these areas, healthcare providers and policymakers can identify effective strategies to improve rehabilitation service accessibility.

Recently, it is evident that scientific research in physical and rehabilitation medicine (PRM) requires careful consideration of study design and methodology. The research question guides the entire research process and helps in selecting an appropriate study design (2). There are three main study designs commonly used in PRM research: descriptive, exploratory/analytic, and experimental. Descriptive designs are used to describe a particular population; exploratory designs aim to explore relationships between variables; and experimental designs investigate the effect of interventions. Epidemiological studies using descriptive or exploratory designs are classified as observational or non-experimental studies. Clinical trials, a type of experimental study, can be classified into different types, such as parallel, crossover, and factorial designs. They can also be categorized based on their purpose, including superiority trials, equivalence trials, and non-inferiority trials. Studies with a higher level of evidence are considered to have a lower risk of methodological bias. Randomized controlled trials (RCTs) are considered the gold standard of design but may not always be feasible in PRM research, especially for non-pharmacological interventions. Difficulties in implementing RCTs lead to the exploration of alternative designs, such as pre-posttest studies and pragmatic trials. Pragmatic trials are designed to evaluate the effectiveness of interventions in real-life clinical settings, while benchmarking controlled trials (BCTs) aim to assess the efficacy of interventions or clinical pathways in observational, real-world settings. These designs are gaining popularity in PRM research due to their better alignment with the complexities of rehabilitation. Proper reporting of study details is essential for transparency and the replication of research. Reporting guidelines, such as STROBE for observational studies and CONSORT for clinical trials, provide frameworks to ensure comprehensive reporting. Efforts, such as the Cochrane Rehabilitation Methodology Meetings and the RCTRACK project, are being made to improve the methodology and generate effective and translational evidence in PRM research (3, 4).

In addition to quantitative research methods, qualitative research methodologies can provide important insights into how interventions work and how patients react to new techniques and methods. In the field of rehabilitation science, the number of qualitative studies published in journals of rehabilitation has increased significantly over the past two decades. There are many areas of opportunity and difficulty include paradigm shifts, advances in methodology, emerging technology, advances in quality evaluation, the growing popularity of mixed-methods approaches, and evolving approaches to knowledge translation. Qualitative research should play an important role in the development of rehabilitation science, and it is crucial that qualitative researchers and methods continue to evolve. The objective of qualitative research in medical rehabilitation is to comprehend the experiences and perspectives of patients and healthcare professionals in relation to medical rehabilitation. It entails collecting data using techniques such as interviews, focus groups, and observation, and analyzing the data to identify themes and patterns (2). Qualitative research can provide valuable insights into the patient experience, including the patient’s beliefs, attitudes, and rehabilitation-related behaviors. It can also assist healthcare professionals in gaining a better understanding of the obstacles and challenges patients face during the rehabilitation process, which can aid in the development of more effective interventions.In qualitative research on medical rehabilitation, frequent topics include patient satisfaction, quality of life, communication between patients and healthcare providers, and barriers to care access (5).

The field of medical and surgical rehabilitation aims to assist individuals in recovering from illness or injury, enhancing their physical function and overall quality of life. Current trends in medical and surgical rehabilitation include the followings: (1) Provide patient-centered care that is tailored to the patient’s specific needs, goals, and preferences. This requires collaborating with patients and their families to develop individualized treatment plans that are geared toward achieving specific outcomes. (2) Utilizing devices and technologies such as virtual reality, robotics, and wearable sensors to aid in recovery and rehabilitation, medical and surgical rehabilitation has become increasingly technologically advanced. These innovations can provide patients with more effective care. (3) Integration of medical and surgical rehabilitation with other healthcare services, such as primary care, mental health care, and social services. This integrated approach aims to improve outcomes by addressing all aspects of a patient’s health and well-being. (4) Professionals from diverse fields, such as physical therapy, occupational therapy, speech therapy, nursing, psychology, and social work, are increasingly collaborating to provide comprehensive care for patients on medical and surgical rehabilitation teams. (5) Prevention by providing patients and their families with information and resources to assist them in maintaining optimal health and avoiding future health problems.

The following are some trends in medical and surgical rehabilitation research:

Personalized Rehabilitation: Personalized rehabilitation is gaining popularity, with an emphasis on individualizing rehabilitation programs based on patient characteristics such as age, gender, climate, environment, and nation. This strategy allows for improved patient outcomes and satisfaction (6).

There is a growing interest in developing less invasive techniques for managing pain, such as the unique technique of mechanical needling with sterile water injection for calcification and fibrosis removal treatment by Areerat Suputtitada (7, 8). This approach can result in less pain, faster recovery times, and a reduced risk of complications.

Transcranial Magnetic Stimulation (TMS) and Transcranial Direct Current Stimulation (tDCS) are two of the most notable noninvasive brain stimulation techniques that have gained significant attention and have shown promise in treating various neurological and psychiatric conditions. TMS and tDCS are both noninvasive methods that can modulate brain activity without the need for invasive procedures. TMS uses magnetic fields to stimulate neurons in the brain, while tDCS uses a low-intensity electrical current. Both methods have shown efficacy in treating conditions such as depression, anxiety, and chronic pain, and ongoing research is exploring their potential in other areas such as swallowing, balance, and gait ability in stroke rehabilitation (9, 10).

Long-term COVID management in post-acute sequelae of SARS-CoV-2 infection (PASC), refers to ongoing symptoms experienced after recovering from acute COVID-19. Rehabilitation is vital in managing this condition, and emerging trends include a multidisciplinary approach, individualized treatment programs, gradual and graded exercise, tele-rehabilitation, and long-term follow-up. It’s essential for healthcare professionals to stay updated as research on post COVID-19 continues to evolve (11, 12).

Tele-rehabilitation is a new trend that uses technology to remotely provide patients with rehabilitation services. Telemedicine may involve virtual consultations, remote monitoring, and teleexercises. This strategy has gained popularity as a result of the COVID-19 pandemic (13, 14).

Virtual Reality: Virtual reality is being used to create simulated environments in which patients can practice real-world activities such as walking, reaching, and grasping. It has been demonstrated that this method increases patient motivation and participation in rehabilitation (13–15).

Robotics: Robotics is used to assist with rehabilitation exercises, such as upper limb exercises following a stroke. Robotic devices can provide a training regimen that is more intensive and repetitive than conventional therapy (13, 14, 16).

Wearable Technology: Smart watches and fitness trackers are being used to monitor and track rehabilitation progress. This method can assist patients and healthcare professionals in identifying improvement areas and monitoring progress (13, 14, 17).

Mind-Body Approaches, such as mindfulness and yoga, are used to supplement conventional rehabilitation programs. These methods can aid in reducing stress and enhancing well-being, which can have a positive effect on rehabilitation outcomes (18).

Intensity of Exercise: Research is increasingly focusing on the optimal intensity of exercise for various populations and conditions. This strategy aims to maximize rehabilitation outcomes while minimizing injury and overexertion risks (19).

Regenerative Medicine: Stem cell therapy and tissue engineering are being studied as potential treatments for conditions that impair the body’s ability to heal, such as spinal cord injuries and osteoarthritis (20).

## Conclusion

In conclusion, rehabilitation plays a crucial role in universal health coverage and the achievement of Sustainable Development Goal 3. It helps individuals of all ages regain independence and improve their quality of life. Qualitative research is essential to understanding the complex experiences and perspectives of patients and healthcare professionals in the field of rehabilitation. Current trends in medical and surgical rehabilitation include personalized care, integration with other healthcare services, technological advancements, interdisciplinary collaboration, prevention, and innovative research areas such as the unique technique of mechanical needling with sterile water injection for calcification and fibrosis removal treatment, noninvasive brain stimulation, long-term COVID management, tele-rehabilitation, virtual reality, robotics, wearable technology, mind-body approaches, optimal exercise intensity, and regenerative medicine. By staying informed and embracing these trends, healthcare professionals can contribute to the advancement of rehabilitation science and provide more effective and patient-centered care.
